# Socioeconomic inequalities in cause specific mortality among older people in France

**DOI:** 10.1186/1471-2458-10-260

**Published:** 2010-05-19

**Authors:** Gwenn Menvielle, Annette Leclerc, Jean-François Chastang, Danièle Luce

**Affiliations:** 1 Inserm U1018, Epidemiology of occupational and social determinants of health, Center for Research in Epidemiology and Population Health, Inserm, Villejuif, France; 2 University of Versailles Saint-Quentin, UMRS 1018, Versailles, France

## Abstract

**Background:**

European comparative studies documented a clear North-South divide in socioeconomic inequalities with cancer being the most important contributor to inequalities in total mortality among middle aged men in Latin Europe (France, Spain, Portugal, Italy). The aim of this paper is to investigate educational inequalities in mortality by gender, age and causes of death in France, with a special emphasis on people aged 75 years and more.

**Methods:**

We used data from a longitudinal population sample that includes 1% of the French population. Risk of death (total and cause specific) in the period 1990-1999 according to education was analysed using Cox regression models by age group (45-59, 60-74, and 75+). Inequalities were quantified using both relative (ratio) and absolute (difference) measures.

**Results:**

Relative inequalities decreased with age but were still observed in the oldest age group. Absolute inequalities increased with age. This increase was particularly pronounced for cardiovascular diseases. The contribution of different causes of death to absolute inequalities in total mortality differed between age groups. In particular, the contribution of cancer deaths decreased substantially between the age groups 60-74 years and 75 years and more, both in men and in women.

**Conclusions:**

This study suggests that the large contribution of cancer deaths to the excess mortality among low educated people that was observed among middle aged men in Latin Europe is not observed among French people aged 75 years and more. This should be confirmed among other Latin Europe countries.

## Background

Socioeconomic inequalities in total and cause specific mortality have been consistently reported throughout Europe [1,2]. Within this general pattern, large differences are observed between countries both in the magnitude of these inequalities and in the weight of different causes of death in inequalities in total mortality. In particular, a specific situation is found regarding cancer mortality. Among men, studies have shown larger inequalities in cancer mortality in France, Belgium and Switzerland. Moreover, the main contributor to inequalities in total mortality was cancer in Latin Europe countries (France, Spain, Portugal and Italy), and cardiovascular diseases in Northern Europe, especially in Nordic countries [1,3]. The picture was less contrasted among women. Moderate inequalities in cancer mortality were found in most European countries except in Spain or Slovenia where no inequalities or even reverse inequalities with higher mortality rates among women with lower socioeconomic position were observed [4]. Also, in women, cardiovascular diseases contributed most to inequalities in total mortality in all countries [1].

Most available studies were conducted among middle aged people and little is known about older people. Fewer studies investigated socioeconomic inequalities in total mortality [5-8] or by cause of death [7,9] among people aged 70 or older. Because mortality rates increase with age, it is expected that relative inequalities will decrease. On the contrary, absolute inequalities that may be quantified using mortality rates difference between educational groups will increase [10,11]. Within this general pattern, there may be variations by cause of death. Studies consistently reported lower but still statistically significant relative inequalities in mortality for total mortality and most causes of death among elderly people when compared with younger people [5-9]. Differences were reported between age groups in the weight of the different causes of death in socioeconomic inequalities in total mortality [7]. Particularly, the contribution of cancer to these inequalities decreased with increasing age. This was found in a pooled European study and thus it still remains unknown whether this decrease was consistently observed in all countries, in particular if it is also observed in countries like France where cancer mortality largely contributes to socioeconomic inequalities in total mortality among middle aged men.

We propose to give some insight to this question by using a French dataset representative of the population. The aim of this paper is to specifically investigate socioeconomic inequalities in mortality by age and causes of death in France, with a special emphasis on men and women aged over 75 and on cancer mortality.

## Methods

We used data from a longitudinal population sample that includes 1% of the French population [12]. We excluded individuals born outside metropolitan France because their vital status was incompletely recorded. Analyses were conducted among three age groups: 45-59 years, 60-74 years, and 75 years and over. For the age group 45-59 years, each subject's follow-up started at 01/01/1990 or his/her 45^th ^birthday, whichever occurred last, and ended at his/her date of death, his/her 60^th ^birthday or 31/12/1999, whichever occurred first. The same applied for the two other age groups. The analysis was conducted among 73374 and 77155 men and women aged 45-59 years in 1990, 51921 and 61895 men and women aged 60-74 years, and 24509 and 41601 men and women aged 75 years and more.

The causes of death were obtained by linkage with the French national death registry (Inserm, CepiDc). The causes of death were identified for 98% of the deceased. We investigated total mortality but also specific causes of death: total cancers (ICD9:140-239), lung cancer (ICD9:162), colorectal cancer (ICD9:153-154), upper aerodigestive tract cancers (UADT) (ICD9:140-150,161), prostate cancer (ICD9:), breast cancer (ICD9:174), cardiovascular diseases (CVD) (ICD9:390-459), ischemic heart disease (IHD) (ICD9: 410-414), cerebrovascular disease (ICD9: 430-438), non cancer-non CVD (ICD9 codes not in 140-239 or 390-459) [13].

The socioeconomic position was measured using education declared at 1990 census and was categorized into four groups: no education, primary education, vocational secondary education, general secondary education and higher.

When quantifying inequalities in different age groups, several methodological aspects should be considered. First, the level of mortality strongly differs between age groups. Therefore, inequalities are best quantified using both relative and absolute measures. A relative measure of inequalities quantifies a ratio whereas an absolute measure quantifies a difference and thus takes into account the absolute level of mortality. An absolute measure of inequalities can be interpreted as an excess mortality among people with low education when compared with people with high education and thus quantifies the burden of the disease in the population. Second, the educational distribution substantially differed by gender and age group. This may have an impact when comparing socioeconomic inequalities between the different groups. Therefore, we quantified inequalities using indices that take into account the size and relative position of each education level and thus minimize problems due to non-comparability in educational distribution between groups compared. We computed relative indices of inequality (RIIs) to quantify relative inequalities and slope indices of inequality (SIIs) to quantify absolute inequality [14,15].

The computation of these indices is based on a relative measure of education. This is a ranked variable that equals, for each educational group, the mean proportion of the population with a higher level of education. Let us consider a population with the following educational distribution: 20% with high education, 30% with middle education, and 50% with low education. For people with high education, this ranked variable is assigned a value of 0.20/2 = 0.10. For people with middle education, it is assigned a value of 0.20+0.30/2 = 0.35. For people with low education, it is assigned a value of 0.20+0.30+0.50/2 = 0.75. The ranked variable was computed separately for each stratum of sex and age category.

The RII was computed using a Cox regression model with this ranked variable as explanatory variable and mortality as the outcome variable. Age was used as time variable. The RII corresponds to the estimate obtained for education (the ranked variable). The SII was then derived from the RII using the following formula: SII = 2*MR*(RII-1)/(RII+1) with MR the mortality rate [1]. Thus, the RII and the SII express inequality within the whole socioeconomic continuum. Both indices use all the available data and are not restricted to comparisons of extreme groups by treating education as a continuous variable. The confidence intervals for both indices were computed with Bootstrap [16].

We also computed age-standardised mortality rates by sex and age group, and by sex, age group and education level, using the 1995 European population as standard. Cause of death certification may be less accurate among the oldest people. Therefore, we conducted additional analyses restricted to the age group 75-84 and thus excluded the very old people whose cause of death may be the least accurately reported. By doing so, we could investigate whether the conclusions drawn for the age group 75 years and more still hold in this restricted group. In addition, we calculated the share of specific causes of death in absolute inequalities in total mortality by dividing the SII observed for a specific cause of death by that found for total mortality.

## Results

**Figure 1:**
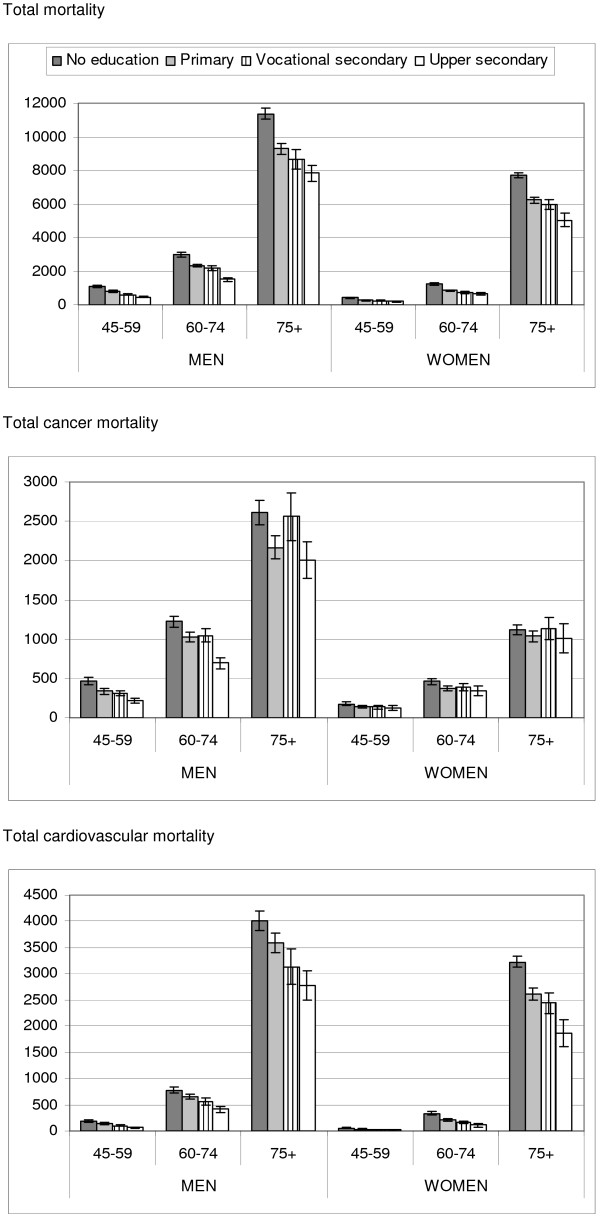
Age-adjusted mortality rate (per 100000 person years) by gender and age group for total and cause specific mortality.

Education level differs by age group and gender (Table 1). The percentage of subjects with an upper secondary or higher education increased from 6% and 14% among women and men aged 75 years and more to 22% and 26% among women and men aged 45-59 years. Cause specific mortality rates differed between age-groups. Among people aged 45-59 and 60-74 years, cancer deaths accounted for about half of total mortality both in men and women (Table 2). Among people aged 75 years and more, CVD were the most prevalent causes of death and accounted for 36% and 42% of total mortality in men and women. A reverse educational gradient was observed for total mortality and CVD mortality for all age groups both in men and women (Figure 1). For total cancer mortality, this gradient was less pronounced among women and it was not observed in the oldest age group for men or for women.

**Table 1:** Education level by gender and age group

	Men			Women		
	
Education level (%)	45-59	60-74	75+	45-59	60-74	75+
No education	20	29	38	22	34	47

Primary education	20	33	37	28	37	36

Vocational secondary education	34	21	11	28	18	11

Upper secondary education or higher	26	17	14	22	11	6

**Table 2:** Relative index of inequality (RII) and 95% confidence intervals (CI) for education, by age group, gender and cause of death

	45-59			60-74			75+		
	
Cause of death	N	RII	95% CI	N	RII	95% CI	N	RII	95% CI
**Men**									
All deaths	2808	3.00	2.50-3.58	7176	2.15	1.92-2.40	10689	1.55	1.41-1.70

All cancers	1291	2.52	1.91-3.26	3165	1.76	1.49-2.08	2630	1.31	1.08-1.57

Lung cancer	362	2.39	1.41-3.89	833	1.68	1.21-2.32	403	1.05	0.66-1.67

UADT cancers	396	4.93	3.06-7.74	506	3.59	2.40-5.65	189	2.95	1.43-6.63

Colorectal cancer	68	1.11	0.33-3.38	261	1.62	0.90-3.00	317	1.51	0.89-2.67

Prostate cancer	17	1.02	0.04-40.4	195	1.02	0.54-1.91	536	1.36	0.90-2.14

CVD	474	3.77	2.40-5.93	1916	2.06	1.67-2.60	3889	1.46	1.24-1.69

IHD	192	2.68	1.40-5.56	767	1.40	1.00-1.96	1087	1.20	0.89-1.62

Cerebrovascular diseases	101	2.23	0.95-5.76	394	2.78	1.79-4.96	989	1.37	1.01-1.88

Non cancer-non CVD	1043	3.37	2.51-4.63	2095	3.07	2.41-3.78	4170	1.85	1.58-2.13

**Women**									
All deaths	1197	2.38	1.78-3.19	3708	2.30	1.96-2.72	15706	1.46	1.34-1.58

All cancers	615	1.57	1.07-2.40	1562	1.44	1.12-1.84	2457	1.07	0.87-1.30

Lung cancer	51	0.69	0.15-2.72	109	0.81	0.30-2.15	126	0.87	0.38-2.11

UADT cancers	38	5.35	0.96-40.1	39	4.39	0.92-28.3	66	0.97	0.26-3.80

Colorectal cancer	55	5.35	1.22-48.1	191	1.25	0.59-2.79	404	0.84	0.52-1.38

Breast cancer	193	1.21	0.60-2.44	333	1.22	0.73-2.09	347	1.26	0.73-2.30

CVD	140	4.63	2.01-11.5	918	3.37	2.43-4.73	6536	1.51	1.33-1.71

IHD	27	3.11	0.50-36.3	270	2.75	1.50-5.37	1422	1.45	1.11-1.92

Cerebrovascular diseases	41	5.98	1.21-54.9	259	2.89	1.62-5.78	1824	1.70	1.33-2.17

Non cancer-non CVD	442	3.55	2.15-5.84	1228	3.24	2.34-4.28	6713	1.60	1.41-1.81

UADT: upper aerodigestive tract groups the cancers of the larynx, oral cavity, pharynx and oesophagus, CVD = cardiovascular diseases, IHD = ischemic heart diseases

The relative inequalities in total mortality as quantified with RII decreased as age increased (Table 2). The RII remained statistically significant among the oldest age group (RII_men _= 1.55, 95% CI: 1.41-1.70; RII_women _= 1.46, 1.34-1.58). Inequalities decreased with increasing age for most causes of deaths. Among men aged 75 and over, the RII was close to 1 for lung cancer and the estimates were not statistically significant for lung, colorectal, and prostate cancer, and IHD. For prostate cancer, the RII increased in the oldest age group. Among women aged 75 and over, the RII remained statistically significant for cardiovascular mortality (RII = 1.51, 1.33-1.71) and non cancer-non CVD causes of death (RII = 1.60, 1.41-1.81) but not any more for cancer mortality (RII = 1.07, 0.87-1.30). For breast cancer, the RII were higher than 1 and similar in all age groups but the pattern by educational level differed between age groups (results not shown). Higher mortality rates were found among less educated women aged 45-59 years and 60-74 years. Among women aged 75 years and more, a U-shape relationship was observed with lower mortality among women with primary or vocational secondary education.

The absolute inequalities as measured with SII increased as age increased (Table 3). This increase was particularly pronounced for CVD. Among men, the SII increased from 138 (per 100000 person years) in the age group 45-59 years to 439 in the age group 60-74 years and to 1340 in the age group 75 years and more. Among women, the figures were respectively 42, 257 and 1168. A large increase was also observed for non cancer-non CVD deaths both in men (from 281 to 698 to 2330) and women (from 115 to 335 to 1353).

**Table 3:** Slope index of inequality (SII) and 95% confidence intervals (CI) for education and mortality rate by age group, gender and causes of death

	45-59			60-74			75+		
	
Cause of death	MR^1^	SII^1^	95% CI	MR	SII	95% CI	MR	SII	95% CI
**Men**									
All deaths	699	699	600 - 788	2356	1724	1485 - 1944	9878	4284	3374 - 5108

All cancers	322	278	201 - 341	1035	572	407 - 724	2363	629	179 - 1054

Lung cancer	90	74	30 - 107	272	138	53 - 216	353	17	-145 - 177

UADT cancers	99	131	100 - 152	164	185	135 - 229	167	165	59 - 246

Colorectal cancer	17	2	-16 - 20	85	40	-9 - 85	288	116	-35 - 262

Prostate cancer	4	0	-9 - 9	66	1	-39 - 41	494	152	-54 - 359

CVD	119	138	98 - 169	635	439	318 - 564	3604	1340	778 - 1854

IHD	48	44	16 - 67	253	84	0 - 163*	995	182	-113 - 469

Cerebrovascular diseases	25	19	-1 - 36	131	123	74 - 174	920	290	12 - 561

Non cancer-non CVD	258	281	222 - 333	687	698	569 - 799	3911	2330	1755 - 2821

**Women**									
All deaths	278	227	156 - 291	957	753	620 - 885	6899	2582	2011 - 3108

All cancers	143	63	10 - 117	403	145	45 - 238	1087	69	-156 - 280

Lung cancer	12	-4	-17 - 11	28	-6	-30 - 21	56	-8	-50 - 40

UADT cancers	9	12	0 - 17*	10	13	-1 - 19	29	-1	-34 - 34

Colorectal cancer	13	18	2 - 25	49	11	-25 - 46	178	-31	-112 - 57

Breast cancer	45	9	-22 - 37	86	17	-27 - 61	154	35	-48 - 121

CVD	33	42	22 - 55	237	257	198 - 309	2867	1168	812 - 1500

IHD	6	7	-4 - 12	70	65	28 - 96	625	228	65 - 393

Cerebrovascular diseases	10	14	2 - 19	67	65	32 - 94	801	413	227 - 591

Non cancer-non CVD	102	115	75 - 145	317	335	255 - 394	2945	1353	996 - 1701

1: per 100000 person-years

MR: age-adjusted mortality rate, UADT: upper aerodigestive tract groups the cancers of the larynx, oral cavity, pharynx and oesophagus, CVD: cardiovascular diseases, IHD: ischemic heart disease

*. 0 included in the CI

No increase in SII was observed for total cancer mortality between the two oldest age groups: the SII remained stable among men from 572 to 629 and decreased among women from 145 to 69, the latter not being significantly different from 0. When investigating the situation by cancer site, the SII decreased among men for lung cancer mortality and did not reach statistical significance in the oldest age group. The SII remained stable and high for UADT cancers. For colorectal and prostate cancer, the SII increased but did not reach statistical significance. In women, the SII remained small and not significant in all age groups for all cancer specific causes of death.

As a consequence of the changes in SII, the contribution of the different causes of deaths to inequalities in total mortality, as quantified by the ratio SII for specific cause of death/SII for total deaths, also substantially differed between age-groups. The contribution of cancer decreased with age from 40% (age group 45-59) to 33% (age group 60-74) and to 15% (age group 75+) among men and from 28% to 19% and to 3% among women. On the contrary, the contribution of CVD increased and reached 31% in men and 45% in women in the oldest age group.

Additional analyses conducted in the age group 75-84 supported the results observed among people aged 75 years and more. Relative inequalities were slightly higher and absolute inequalities slightly smaller than those found in the age group 75 years and more.

## Discussion

### Summary of main findings

We investigated educational differences in mortality by age group and especially among the oldest (age 75+). Relative inequalities were still observed in the oldest age group although the magnitude decreased when age increased. Absolute inequalities increased with age. The share of different causes of death to absolute inequalities in total mortality differed between age groups. In particular, the contribution of cancer deaths decreased substantially between the age group 60-74 years and the oldest age group. Thus, the specific "Latin Europe" pattern characterised by an important contribution of cancer deaths to the excess mortality found among low educated middle aged men was not observed among people aged 75 years and more in France.

### Methodological aspects

While we used a high quality dataset made of 1% of the French population and including institutionalised persons, some limits of the data should be addressed. The choice of the socioeconomic position indicator should be discussed. Education presents several advantages, in particular it is available both among men and women and also among older people, but also drawbacks [17]. First, the meaning of education is related to age. The measures we used to quantify socioeconomic inequalities are especially well adapted to compare populations with different education distributions and thus partly account for this issue. In addition, education is determined early in adulthood and may not any more accurately describe the socioeconomic position of old people. It has been suggested that economic advantage or disadvantage accumulated over the life course as measured by long-term income or total financial assets may better capture the cumulative effects of lifetime socioeconomic position on health status in old age and may thus be more appropriate when studying older populations [18,19]. This information was not available in our dataset. Moreover, as we aim to study changes in inequalities with age, we had to use an indicator that was adapted to different age groups. In that sense, while not being the most adequate indicator for elderly people, education is a good indicator as it has been shown to be consistently associated with health through age groups and gender.

The mechanisms through which education may impact health are diverse [20]. In addition to consequences of education in terms of lifetime socioeconomic position, people with higher education may be more receptive to prevention messages and may have a better ability to change their health behaviour and to better use the health care system. Among elderly people, factors in relation with health care may be the most relevant. This includes more regular visits to GPs and specialists, a higher uptake of screening, a better understanding of the prevention messages or a better compliance to treatment among more educated people.

Some problems may occur with cause of death certification, especially among the oldest people. If so, the results observed in the oldest age group may be, at least partly, due to problems in cause of death certification rather than to real changes in the mechanisms of socioeconomic inequalities. Then, CVD may be more easily coded among old people certainly suffering from multiple diseases which could lead to death. To explore this hypothesis, we investigated the distribution of the associate causes of death, which are the causes of death mentioned on the death certificate in addition to the underlying and the immediate cause of death. In the oldest age group, the associate causes of death for CVD deaths were also CVD for 25% of deaths, non cancer-non CVD otherwise. The associate causes of death for cancer deaths were mainly non cancer-non CVD. This pattern did not substantially differ by education. These findings did not suggest that CVD would be more easily coded among older people. Moreover the percentage of causes of death unknown or ill-defined was small (around 3%) and similar in all age groups stratified by sex and education. We also conducted analyses restricted to people aged 75-84. Results were consistent with those observed in the broader age group 75 years and more. The largest difference was found for non cancer-non CVD deaths, suggesting that, if any, imprecision in cause of death certification would favour a broad and heterogeneous group of diseases (non cancer-non CVD) rather than CVD. Thus, we do not believe errors in cause of death certification substantially biased our results.

External causes of death were included in the group non cancer-non CVD. When studied separately, the inequalities for external causes of death were globally similar to those found for non cancer-non CVD deaths, although based on small numbers of deaths.

Our findings reflect both cohort and age effects. We quantified inequalities using indicators (RII and SII) that were well adapted to compare populations with different educational distribution and thus partly accounted for the cohort effect. Because individuals aged 75 years and more experienced specific life events that younger individuals will not, for instance the First World War, a partly different pattern is likely to be observed in younger cohorts.

### Decrease in relative inequalities with age

Decrease in relative inequalities in mortality with age, for total mortality as well as for cause specific mortality, has already been reported in the literature [5,7,21] and is partly due to a selection effect. Individuals with lower socioeconomic status may die earlier, so that only the healthiest survive into old age, leading to reduced socioeconomic inequalities in mortality. We could then wonder whether socioeconomic inequalities totally disappeared among very old people. A study suggested that, despite a selection effect, socioeconomic inequalities in total mortality in France persist till very old age: inequalities were not any more observed after age 94 [22]. In addition to the selection effect, the decrease in relative inequalities may also partly be explained by the attenuation of the association between risk factors and chronic disease at old age [23].

We observed differences between gender and age groups that may be specific to the French situation. For lung cancer, we found higher mortality rates among higher educated women and among lower educated men, especially among younger men. Among men, we can see the timing of the smoking epidemic with progressively higher smoking rates and consequent higher lung cancer rates among lower educated men. These results reflect the smoking epidemic which is in France at an earlier stage than in Northern European countries [24,25]. For breast cancer, we did not observe any clear association between breast cancer mortality risk and education. Explanations for this finding have already been discussed [26] and include changes in the fertility pattern (mainly age at first birth) and improvement in treatment and screening during the last decades in France. In most countries, higher mortality rates are reported among higher educated women in most European countries [27,28]. These inequalities may nevertheless decrease in future years also in other countries as suggested by the weaker association between education and breast cancer mortality reported among younger women in several countries [27].

### Increase in absolute inequalities with age

Increase in absolute inequalities with age has also been reported before [6-8]. The present study documented for the first time inequalities among older people in France, where large inequalities in cancer mortality and an important and increasing contribution of cancer to inequalities in total mortality had been reported, especially among middle aged men [29]. The present study showed that this specific situation does not remain among older people. In this age group, the pattern is more comparable to what is found in Nordic countries and the UK with a large contribution of cardiovascular diseases and a decrease in the contribution of cancer [1,3].

Interestingly, whereas absolute inequalities in total mortality increased with age, there was no increase in male absolute inequalities for cancer mortality between the two oldest age groups and we reported a decrease in female absolute inequalities for cancer mortality between these two age groups. This contrasts with the available literature that shows an increase in absolute inequalities for all causes of deaths, including cancer mortality [7]. The explanations for this decrease are not straightforward but several could be suggested. The magnitude of mortality rates may be part of the explanation. Absolute inequalities combine both the level of mortality and the magnitude of relative inequalities. Relative inequalities were only slightly larger for CVD mortality than for cancer mortality among the oldest men. The differences in absolute inequalities are thus mainly driven by variations in mortality rates. In France, cancer mortality rates are especially high among middle aged men but not any more among older people when compared with other European countries. If we cannot rule out the possibility that cancer is more often used as the underlying causes of death in France [30], it is not clear why this would mostly affect younger people. It has been suggested that differences between countries in socioeconomic inequalities in the distribution of risk factors accounted for the differences observed in socioeconomic inequalities between countries. Thus, the pattern of socioeconomic inequalities in France would be partly due to large socioeconomic inequalities in alcohol consumption [31,32] as well as lower rates and modest socioeconomic inequalities for overweight and obesity [33] or smoking [24,25]. This may be especially visible at younger age, when the incidence rate of the chronic disease associated with these risk factors peaked. This may partly explain why mortality rates and socioeconomic inequalities are much higher for cancer than cardiovascular disease among middle aged people. At older age, this specific situation may disappear partly due to selection effect combined with an attenuation of the association between risk factors and chronic diseases at old age.

In a context of ageing population, the share of elderly people among the general population is currently increasing. Because the pattern of socioeconomic inequalities in mortality, and especially the contribution of different causes of death to these inequalities, is likely to differ by age, efficient public health policies aiming at decreasing these inequalities should be defined for each age group. More particularly, the share of cancer deaths to absolute inequalities in total mortality decreased among older French people. In France, reducing socioeconomic inequalities in cancer was recently identified as a main target for public health policies for the next years. Our results show that, if successfully implemented, public health policies focusing on cancer will substantially reduce inequalities before age 75 in France. They may have comparatively little effect among elderly people. If we want to be successful in decreasing inequalities among older people in France, our results suggest that policies should mostly concentrate on cardiovascular diseases.

## Conclusions

European comparative studies documented a clear North-South divide in socioeconomic inequalities in mortality. They reported a large contribution of cancer deaths to the excess mortality among low educated middle aged men in France and other Latin countries contrary to Northern Europe countries. Our results suggest that this large contribution of cancer deaths is not any more observed among older French men and women. The pattern of socioeconomic inequalities by cause of death among elderly people may thus not largely differ between European countries contrary to what is found at younger age. This should be confirmed with data from other Latin Europe countries.

## Competing interests

The authors declare that they have no competing interests.

## Authors' contributions

GM conducted the analysis and wrote the draft. AL, JFC and DL discussed the analyses and the results and commented on the manuscript. All authors approved the final version of the manuscript.

## Pre-publication history

The pre-publication history for this paper can be accessed here:

http://www.biomedcentral.com/1471-2458/10/260/prepub
